# Evolutionary Epidemiology of Drug-Resistance in Space

**DOI:** 10.1371/journal.pcbi.1000337

**Published:** 2009-04-03

**Authors:** Florence Débarre, Thomas Lenormand, Sylvain Gandon

**Affiliations:** Centre d'Écologie Fonctionnelle et Évolutive, CNRS – UMR 5175, Montpellier, France; University of California San Diego, United States of America

## Abstract

How can we optimize the use of drugs against parasites to limit the evolution of drug resistance? This question has been addressed by many theoretical studies focusing either on the mixing of various treatments, or their temporal alternation. Here we consider a different treatment strategy where the use of the drug may vary in space to prevent the rise of drug-resistance. We analyze epidemiological models where drug-resistant and drug-sensitive parasites compete in a one-dimensional spatially heterogeneous environment. Two different parasite life-cycles are considered: (i) direct transmission between hosts, and (ii) vector-borne transmission. In both cases we find a critical size of the treated area, under which the drug-resistant strain cannot persist. This critical size depends on the basic reproductive ratios of each strain in each environment, on the ranges of dispersal, and on the duration of an infection with drug-resistant parasites. We discuss optimal treatment strategies that limit disease prevalence and the evolution of drug-resistance.

## Author Summary

The spread of drug-resistant parasites erodes the efficacy of therapeutic treatments against many infectious diseases and is a major threat of the 21st century. The evolution of drug-resistance depends, among other things, on how the treatments are administered at the population level. “Resistance management” consists of finding optimal treatment strategies that both reduce the consequence of an infection at the individual host level, and limit the spread of drug-resistance in the pathogen population. Several studies have focused on the effect of mixing different treatments, or of alternating them in time. Here, we analyze another strategy, where the use of the drug varies spatially: there are places where no one receives any treatment. We find that such a spatial heterogeneity can totally prevent the rise of drug-resistance, provided that the size of treated patches is below a critical threshold. The range of parasite dispersal, the relative costs and benefits of being drug-resistant compared to being drug-sensitive, and the duration of an infection with drug-resistant parasites are the main factors determining the value of this threshold. Our analysis thus provides some general guidance regarding the optimal spatial use of drugs to prevent or limit the evolution of drug-resistance.

## Introduction

The widespread use of antimicrobial drugs during the 20^th^ century greatly contributed to the increase in human life expectancy [1]. Yet, the emergence and the spread of drug resistant parasites erode the benefits associated with these treatments [2]. We now face the challenge of “resistance management”, which consists in finding a treatment strategy that most reduces the number of infections, while keeping drug-resistance at a low frequency. We therefore have to use optimized treatment policies [3].

The development and the analysis of mathematical models have played a major role in our understanding of the evolutionary dynamics of drug resistance. These epidemiological models allowed to compare various treatment strategies such as different treatment doses [4],[5], the mixing of different drugs, or treatment cycling [3],[6]. However, most of these theoretical studies focused on the evolution of resistance in a single isolated population. The evolutionary dynamics of drug resistance in a spatially heterogeneous environment seems to have been largely overlooked in the context of infectious diseases in humans. Yet, attempts have been made to take into account some aspects of spatial heterogeneity using spatially implicit models. In these models, the host population is structured into different compartments experiencing different treatment strategies [7]–[9]. The importance of migration rates among different compartments was pointed out, but relied on a simplified description of the spatial spread of parasites, with no isolation by distance. Such a metapopulation framework [10] is well suited to model the evolutionary dynamics of drug resistance in networks of hospitals [9] but fails to capture situations with spatially limited dispersal.

The issue of resistance management is however not restricted to human infectious diseases. For example, drug-resistance decreases treatments efficiency in livestock [11], compromises the control of parasitic fungi and pests in conventional [12] and genetically modified crops [13]. The impact of the spatial heterogeneity of the environment has been studied in models of fungicide resistance [14],[15], but also in models of insecticide-resistance management [16]–[19], with the concept of a “stable zone strategy” [18], where a heterogeneous treatment lowers the density of pests, while preventing the onset of resistance, provided the treated area is below a critical width.

The underlying concept comes from population genetics studies [20]–[24] on the persistence of an allele under spatially varying selection. When the favorable zone is smaller than a critical size, migration counteracts the effects of natural selection, and gene swamping occurs [25]. In these analytical studies however, the population parameters (including the carrying capacity) are arbitrarily fixed (but may vary in space [24]). In an epidemiological context, the total parasite density, evaluated via the total density of infected hosts, is typically not constant. Thus, we extend in this paper the concept of a critical treatment area to an epidemiological setting. In particular, we focus on the interplay between demography – the total density of parasites – and the frequency of drug-resistance. We consider two kinds of disease transmission: (i) by direct contact between infected and non-infected individuals, and (ii) via dispersing vectors. Under these different scenarios, we discuss optimal treatment strategies that prevent or limit the evolution of drug-resistance in a linear environment.

## Results

### Direct transmission model

We first study a parasite life-cycle with direct parasite transmission between hosts. At time 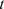, and at each point 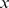 in a one-dimensional environment, the host population is divided into uninfected individuals, 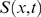, infected individuals that carry drug-sensitive parasites (labeled 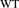, for wild-type, throughout the paper), 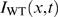, or drug resistant parasites (labeled 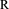 throughout the paper), 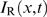. The total density of infected individuals is thus 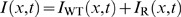, and the proportion, among all infected individuals, of individuals infected by drug-resistant parasites is 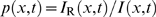. The within-host parasite dynamics is not explicitly modeled, and 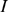 and 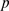 will hereafter be referred to as the total parasite density and the frequency of resistant parasites, respectively.

**Figure 1:**
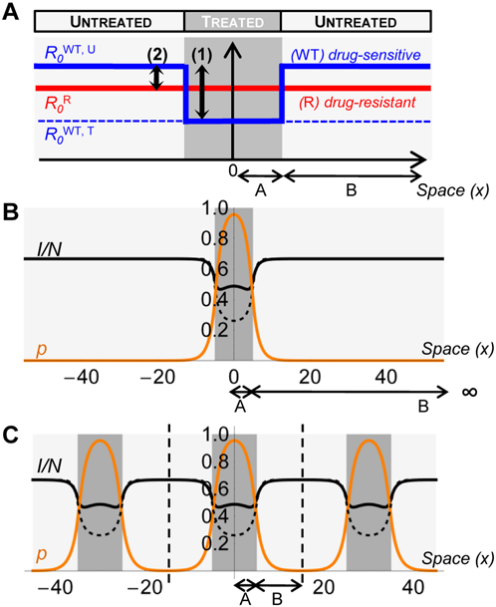
Effects of spatial heterogeneity on the parasites. The treated area is represented with a dark gray filling. Subfigure (a) shows the effects of treatment on the basic reproductive ratios 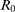. Blue stands for drug-sensitive and red for drug-resistant. The arrow (1) shows the effect of treatment on the drug-sensitive parasites, and (2) the cost of drug-resistance. Subfigures (b) and (c) show numerical resolutions of the direct transmission model. In (b), the width of the untreated area, 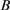, is infinite; in (c), 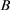 is finite. In (b) and (c), the full black curve is the prevalence of the disease at equilibrium, 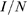, and the orange curve is the cline of frequency 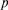 of individuals infected by the drug-resistant strain, among all infected individuals. The dashed black curve represents the equilibrium prevalence in the absence drug-resistant parasites. Parameters: 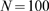, 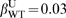, 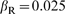, 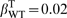, 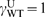, 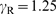, 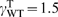.

The disease is transmitted locally by direct contact between an infected and a susceptible individual, with a transmission parameter 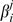. The subscript 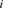 refers to the parasite type: 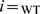 for drug-sensitive parasites and 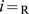 for drug-resistant parasites; the superscript 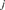 refers to the area type: 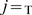 in the treated area, and 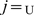 in the untreated area. Note that no superscript 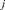 is required for drug-resistant parasites, since they are not affected by the treatment, see figure 1. Infected individuals recover at rate 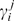, which corresponds to parasite clearance. Recovered individuals immediately become susceptible to the disease again, as in classical SIS models [26]. Finally, the total density of the host population remains constant in space and time (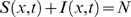). This assumption implies that models with frequency-dependent or density-dependent selection both lead to the same results [27]. Note also that, even though the total host density 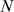 is held constant, the prevalence of the infection, 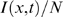, varies in space and time.

The environment is linear, and divided into treated and untreated areas, of width 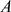 and 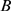, respectively. We focus on simple spatial patterns of treatment: a pocket of treatment in an infinite untreated region (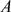 small compared to 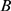, see figure 1b); or a periodical zebra-like pattern of treated and untreated regions (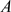 and 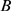 of the same order of magnitude, see figure 1c). All infected individuals are treated in the treated area (but our model can be readily extended to allow for a partial treatment). The treatment lowers the transmission of drug-sensitive parasites (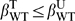), and/or increases their clearance (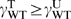), while drug-resistant parasites remain unaffected. The resistance allele induces a fitness cost [28], so that drug-resistant parasites are selected for in the treated area, but selected against in the untreated area. This can be seen by comparing the basic reproductive ratios, 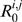, of the two strains (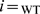 or 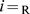) in the two different environments (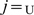, for untreated, 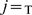, for treated; see figure 1a, and equation (1) below). The basic reproductive ratio 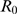 is a compound parameter in epidemiology, defined as the total number of secondary cases due to the introduction of a single infected individual in a susceptible population [26],[29],[30]. In a well-mixed population, a disease will spread only if 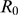 is above unity [31]; 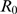 is also used to compare different parasites, and to predict pathogen evolution [32]. For strain 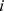 in habitat 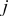, we have:
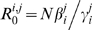
(1)


As illustrated on figure 1a, the drug-resistant parasites have a higher basic reproductive ratio than the drug-sensitive parasites in the treated area (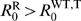). By contrast, the drug-sensitive parasites have a higher basic reproductive ratio than the drug-resistant ones in the untreated area (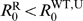).

Both infected and uninfected hosts migrate. The distribution of the distances of migration (i.e. the kernel of migration) of both hosts is assumed to be symmetric (i.e. with mean 0) and with variance 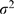. We use the classical diffusion approximation, and higher moments of the distribution are therefore neglected [33]. The above assumptions results in a system a partial differential equations for the density of people infected by the drug-sensitive (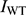) and drug-resistant parasites (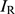), which is presented in the Materials and Methods section (see system 14). We derive from that system the following dynamical equations for the epidemiology (parasite density 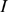) and the evolution (frequency of resistance 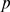) of the parasite population at the point 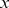 and time 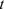 (for readability we drop the time and space dependence notation in 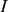 and 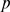):
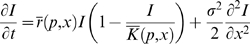
(2a)

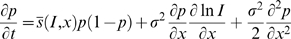
(2b)where 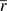, 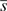 and 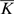 are (using 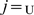 in the untreated area and 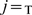 in the treated area):
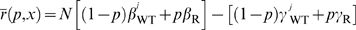
(3)

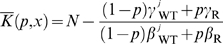
(4)

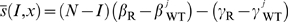
(5)These variables can be interpreted as a frequency-dependent population growth rate (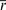), frequency-dependent population carrying capacity (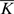), and density-dependent selection coefficient for drug-resistant parasites (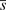).

This formulation clarifies the feed-back of demography on evolution (i.e. the selection 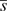 varies with the prevalence of the infection 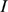), and vice versa (i.e. the parasite population growth rate 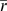 depends on the frequency of resistance 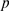). Figures 1b and 1c show examples for the spatial variation in prevalence (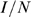) and in the frequency of resistance (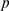) at equilibrium.

Following on earlier studies [22],[23],[34], we derived the exact minimal size of the untreated area, 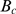, for drug-sensitive parasites to invade a drug-resistant parasite population (see Text S1). The opposite case, namely the invasion condition for a drug-resistant strain to invade a drug-sensitive population, is more complicated. Indeed, while the drug-resistant parasites' traits are constant in space (as we assume that the treatment has no effect on them), the drug-sensitive parasites' transmission and recovery parameters depend on the spatial location. As a result, the equilibrium density of a parasite population fixed for the drug-sensitive type varies across space, and we did not find an exact analytic expression for this equilibrium density (but approximate solutions can be found using perturbation solutions [35]). This prevents us from deriving a general invasion condition for drug-resistance. Yet, we present below this invasion condition for two extreme migration scenarios.

First, when the migration range is restricted to the nearest neighbors (i.e. 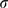 is small compared to 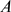 and 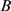), the density of the drug-sensitive parasite population varies sharply between the treated and untreated areas. We assume that 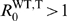, i.e. that the drug cannot totally eradicate the parasite in a well-mixed population even when all individuals are treated (the results with a more efficient treatment, such as 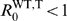, are presented in Text S1). The effect of the initial asymmetry in population size can be approximated by 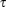, the (untreated/treated) ratio of the equilibrium parasite densities in each area in the absence of migration:
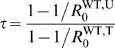
(6)Let us define 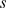 and 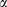, so that 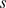 (resp. 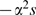) is the drug-resistant parasite's initial rate of increase in a wholly treated (resp. untreated) well-mixed population fixed for the drug-sensitive parasite. Using equation (5), and rearranging, we obtain:
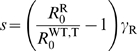
(7a)

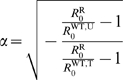
(7b)Using this notation, the critical width of the treated area, under which the drug-resistant strain cannot invade when initially rare, reads:
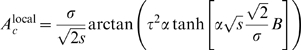
(8)which is similar to the result found in earlier population genetics studies [24], in which 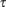, 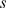 and 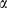 are fixed parameters values. In contrast, our study allows demography to feed-back on evolution; the population parameters (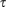, 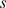, 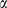) are interdependent and vary with the underlying life-history traits (see equations (6) and (7)).

Equation (8) shows that the critical size 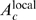 is proportional to the range of migration 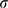: the further the hosts migrate, the more difficult it is for the drug-resistant parasites to invade. A high migration indeed reinforces gene swamping. This recalls a classical result in island models, which is that migration might prevent the maintenance of diversity [36]–[38]. Equation (7a) shows that the direction of selection is determined by the basic reproductive ratios 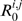 of each strain in each environment. In other words, as in classical well-mixed models (see equation (10) below), the basic reproductive ratios summarize most of the heterogeneity in selection pressures acting on the two parasite types. Yet, an additional epidemiological parameter, the recovery rate from an infection with drug-resistant parasites, 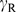, is required to determine the invasion condition of the drug-resistant parasites. In a spatially heterogeneous environment indeed, the fate of drug-resistant parasites does not only depend on the *direction* of selection (governed by the ratio of 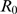
s), but also on the *intensity* of selection in the two environments. The intensity of selection is inversely proportional to the drug-resistant parasites generation time, 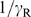.

In contrast, when there is very long-range migration (high 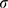 compared to 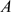 and 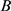), the effects of treatment can be averaged over the whole habitat and, consequently, the asymmetry in population sizes among treated and untreated areas can be neglected. A classical invasion analysis based on the calculation of the basic reproductive ratios of the different parasites is used to derive the critical area size:
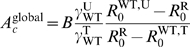
(9)Note that this critical size depends not only on the various basic reproductive ratios (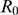), but also on the ratio of the recovery rates from an infection by the drug-sensitive strain, in the untreated (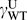) and treated (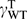) areas.

Instead of treating every infected individual in a restricted area corresponding to a proportion 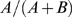 of the environment, we can choose to treat everywhere the same proportion 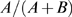 of infected individuals. We refer to this strategy as a homogeneous treatment. Under this homogeneous strategy, the critical 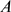 only depends on the basic reproductive ratios:
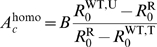
(10)Hence, if we assume that the treatment increases the recovery rate (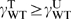), then 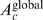 is smaller than 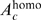. At high migration, drug-resistance therefore appears more easily with a heterogeneous treatment than with a homogeneous treatment.

**Figure 2:**
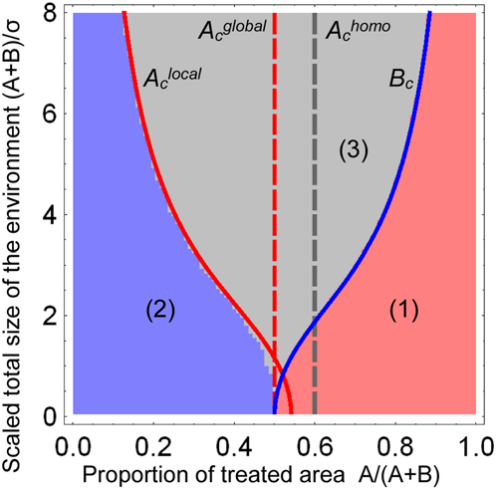
This Reciprocal Invasion Plot represents the outcome of the competition between drug-resistant and drug-sensitive parasites (direct transmission model). This outcome depends on the total size of the environment scaled by the migration range parameter 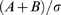, and on the proportion of the treated area 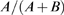. The curves show the analytical predictions, and the surfaces result from numerical integrations. The curves delimit regions in the parameter space where a parasite type can invade a population fixed for the other type. Both types coexist when each type can invade the other (i.e. when there is reciprocal invasion). The dashed red curve is obtained from the critical size 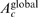 (see equation (9)); the full red curve corresponds to 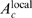 (see equation (8)), the full blue curve is obtained from 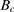 (see Text S1), and finally the dashed gray curve comes from 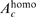, corresponding to a spatially homogeneous treatment (see equation (10)). In the red zone (1), only the drug-resistant strain persists at equilibrium; in the blue zone (2), only the drug-sensitive strain persists; in the gray zone (3) both strains coexist at equilibrium. Parameters: 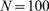, 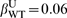, 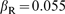, 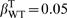, 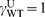, 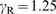, 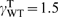.

Figure 2 combines the above analytical results in a reciprocal invasion plot, together with the outcome of numerical integrations of system (2). For a fixed set of epidemiological parameters, we explore the possible outcomes of the system, depending on the scaled total size of the environment 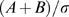 and the proportion of the population that receives treatment (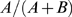) (Figure S1 shows the same results, but plotted in function of the scaled sizes of the untreated (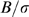) and treated (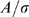) areas). With our parameters, three outcomes are possible at equilibrium: exclusion of the drug-sensitive strain (zone (1) in figure 2), exclusion of the drug-resistant strain (zone (2)), or coexistence of both strains (zone (3)). With other parameters, a fourth situation is possible, corresponding to evolutionnary bistabilities, where only one strain is maintained, its type depending on the initial conditions (see Figure S2). Figure 2 confirms that 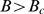 is an exact invasion criterion for the drug-sensitive strain, while 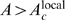 and 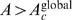 give good approximations of the invasion criteria of the drug-resistant strain for low and large migration ranges 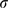, respectively.

### Vector-borne transmission model

In the above section we focused on a scenario with parasite transmission by direct contact among hosts. In the following we consider a more complex parasite life cycle involving two different host species. In particular, we focus on vector-borne transmission such as in malaria, leshmaniosis, trypanosomiasis and many other human infections (the model holds for any disease involving the sequential infection of two different hosts, and can be readily extended to other two-stage life-cycles, with air-borne or water-borne transmission for instance). Hereafter, we call the first host “human”, and the second host “vector”. Both humans and vectors can migrate, though at potentially different ranges (with parameters 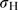 and 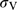 respectively); the humans recover (or die) at rate 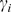 (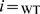 for the drug-sensitive strain, and 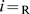 for the drug-resistant strain), and the vectors disappear at rate 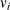. The total densities of humans (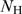) and vectors (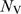) remain constant, but the prevalence of the infection may vary. In order to determine the critical width of the treated area, under which the drug-resistant parasites cannot invade, we use a low-migration approximation as in the previous section. The asymmetries in population sizes between untreated and treated areas are measured by the ratios 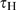 (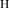 for humans) and 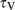 (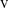 for vectors) (see Text S1 for their formulation).

**Figure 3:**
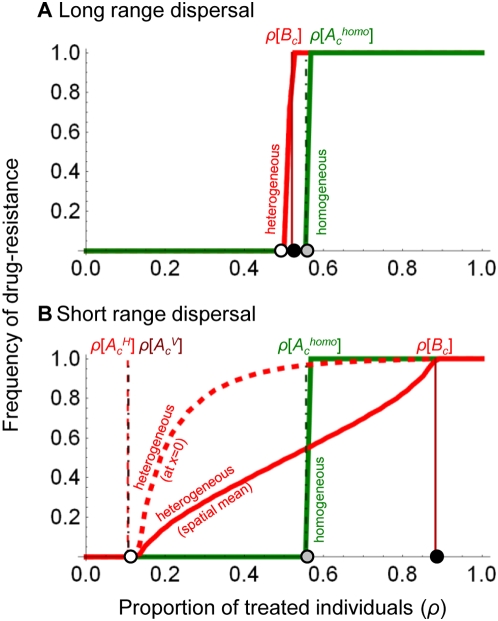
Comparison between homogeneous and heterogeneous treatments. This figure represents the frequency of drug-resistance at equilibrium, as a function of the proportion of treated individuals (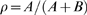), for the homogeneous (green) and heterogeneous (red) treatment strategies, with the vector-borne transmission model. The migration range is high in (a), and low in (b). The curves result from numerical integrations of the model, and the vertical lines show the analytical predictions; 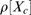 means “proportion of treated individuals corresponding to the critical size 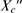
”. The red full curve shows the (spatial) mean frequency of drug-resistance, while the dashed red curve shows this frequency at the center of the treated area (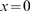). Parameters: 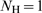, 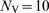, 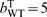, 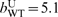, 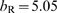, 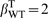, 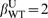, 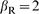, 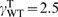, 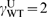, 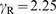, 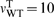, 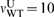, 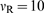; in (a) 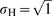, 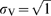; in (b) 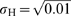, 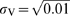.

We find two critical sizes 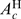 and 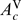, depending on whether the initial density is calculated in the human (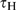) or vector (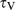) compartments. Simulations show that these two critical sizes closely bound the real critical size (see figure 3b). With 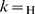 or 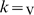, these bounding critical sizes read:
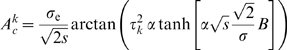
(11)The equivalent migration range 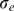 depends on the humans' and vectors' migration ranges, but also on the duration of the infection in both humans and vectors, and reads:
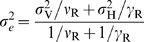
(12)The selection parameters 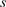 and 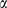 are such that 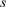 (resp. 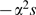) is the intensity of selection for the drug-resistant parasite in a well-mixed wholly treated (resp. untreated) population fixed for the drug-sensitive strain. After rearranging, we obtain:
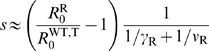
(13a)

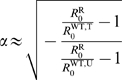
(13b)(see Text S1 for the whole expression for the basic reproductive ratios 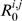).

As in the single-host life cycle, the fate of drug-resistant parasites depends on the intensities of selection in treated (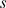) and untreated (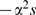) areas. We thus recover very similar expressions for the intensity of selection (compare equations (7) and (13)), which depend on the ratios of 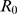 s and on the generation time of the drug-resistant strain, which is 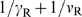 in the two-host model.

The drug-sensitive invasion condition, 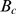, is presented in Text S1. The homogeneous invasion condition (see equation (10)) holds, provided that the basic reproductive ratios are modified according to the new life-cycle.

## Discussion

In this study, we analyze the interplay between epidemiological and evolutionary dynamics of a drug-resistant parasite strain in a one-dimensional environment. Following on and extending earlier population genetics studies on clines [22]–[24], we derive approximations of invasion conditions for drug-resistant and drug-sensitive strains, and for different modes of parasite transmission (by direct contact, or vector-borne). In particular, we derive a critical treatment area size below which a drug-resistant strain cannot invade a population fixed for the drug-sensitive strain. Under the critical treatment size, the effects of gene flow (i.e. the immigration of drug-sensitive parasites from untreated areas, into the treated) are stronger than the effects of natural selection (which favors the drug-resistant strain in the treated area). Understanding the factors that govern the value of these critical treatment areas has direct practical implications: in particular, it may allow one to optimize the use of antimicrobial drugs to prevent the emergence and spread of drug-resistant pathogens. Furthermore, in the broader context of insecticide-resistance, fungicide-resistance, or resistance to toxins in genetically modified crops, taking space into account may help develop new resistance management strategies.

In our direct transmission model, as pointed out earlier by Nagylaki [22],[24] in a population genetics context, the critical size of the favorable area is proportional to 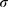, the standard deviation of the distribution of the distances of migration (i.e. the standard deviation of the migration kernel), which is thus a measure of the migration range. More migration increases the critical size because it counteracts the effect of natural selection in the treated area.

Second, as emphasized by Nagylaki [24], asymmetric densities (summarized in the compound parameter 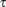) generate asymmetric gene flow that selectively favor the allele in the most populated area. In Nagylaki's study [24], 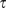 and the selection parameters 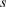 and 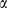 are independent. In our study however, the population parameters (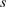, 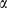, 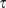) depend on explicit individual life-history traits (such as 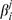, 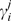) [39] (for the direct transmission model, see equations (6), (7a) and (7b) for 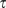, 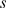, and 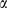). Consequently, in contrast to earlier population genetics studies [24], the effects due to the asymmetry in population sizes between habitats (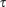) and to the heterogeneity of selection pressures (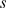, 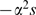) are intermingled. In addition, 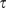 is always greater than unity. Our critical size is therefore greater than when no epidemiological feedback on evolution is considered (see Text S1 for a comparison between models with or without demographical feedback). The initial asymmetry in drug-sensitive parasites' densities makes the drug-resistant parasites' invasion harder.

A third factor determining of the critical size is the intensity of selection for the invading strain, in each environment (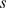 and 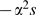). In a spatially homogeneous habitat, where the intensity of treatment does not vary in space, the invasion conditions are exclusively governed by the sign of 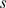, which only depends on the basic reproductive ratios, 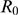, of the different parasites in treated and untreated areas (see 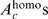 expression in equation (10)). Yet, in a spatially heterogeneous environment, where treatment varies in space, in addition to its *direction*, the *intensity* of selection in both areas is required. The intensity of selection is inversely proportional to the total duration of an infection with the drug-resistant strain (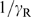 with the direct transmission model, and 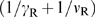 with the vector-borne transmission). This explains the impact of the drug-resistant infection duration on the critical area size. As a result, for a given value of 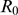, the shorter the duration of the infection (i.e. high 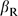 and 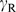), the more likely is the drug-resistant parasite's invasion (which corresponds to a lower critical 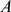 size, see equations (8) and (11)), because the hosts have less time to leave the favorable area. Consequently, a parasite with fast dynamics is better locally adapted that one with slower dynamics.

The basic reproductive ratios are classically used in epidemiological models to evaluate the costs of drug-resistance [40],[41], as we did in figure 1a. Here we show that it is critical to know which life-history traits are affected in drug-resistant parasites. This point has already been raised in models with temporally varying environments [42],[43]. Thus, both temporal [43] and spatial heterogeneities have the potential to alter the conclusions of models of well-mixed populations, because the direct correspondence between 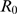 and the fitness of a parasite strain does not hold anymore. A practical implication of our results is that accurate predictions regarding the evolution of drug-resistance require more information on the life history traits of the parasites which contribute to the cost of drug resistance [44].

### Resistance management

Our model can be used to explore new strategies of resistance management. Yet, various criteria can be used to define an optimal strategy [45],[46], based on the short-term or long-term minimization of parasite prevalence, or on the frequency of drug-resistance at equilibrium or in a transitory phase. In our model, we focus on the equilibrium (i.e. long-term) frequency of drug-resistance across space. We define a good treatment strategy as a strategy under which a maximum proportion of individuals can be treated, but which best prevents or limits the emergence and spread of drug-resistant parasites.

Suppose that only a limited stock of treatment is available: only a part of the population can be treated. Two (extreme) strategies are considered: treating everyone in a limited area of width 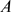, the total size of the environment being 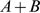 (a strategy referred to as heterogeneous treatment), or treating the same proportion 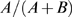 of individuals everywhere (homogeneous treatment). In both strategies, the same overall number of individuals are treated, and all of them receive the same dose of treatment. Note that the homogeneous treatment is homogeneous from a global perspective, but heterogeneous locally (and conversely for the heterogeneous treatment). Our definition thus contrasts with the terminology used by other authors (e.g. [6]). Which treatment strategy best prevents the invasion of drug-resistant parasites? With a homogeneous treatment, there is no coexistence at equilibrium, and the population will either be fixed at equilibrium for drug-sensitive parasites, or for drug-resistant parasites. With a heterogeneous treatment, however, drug-sensitive and drug-resistant parasites can coexist at equilibrium. We compare the evolutionary outcomes obtained under the two treatment strategies in figure 3, using our model of vector-borne transmission.

For our set of epidemiological parameters, when the migration range is large (figure 3a), three different cases may be considered: (1) when the proportion of treated individuals is low (on the left-hand side of the white point in figure 3a), both strategies are equivalent because drug resistance does not emerge; (2) when the proportion of treated individuals is intermediate (between the white and gray points), drug resistance emerges and spreads in the heterogeneous treatment strategy but not in the homogeneous one; (3) when the proportion of treated individuals is high (on the right-hand side of the gray point, so that 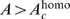), both treatment strategies are equivalent since drug resistance spreads to the whole population under both scenarios.

When migration is more local (figure 3b), drug-resistance still appears for a smaller proportion of treated individuals with the spatially heterogeneous treatment. However, when the proportion of treatment is such that 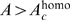 and 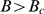 (i.e. between the gray and black points in figure 3b), drug-resistant parasites dominate the whole environment with the homogeneous strategy, while the heterogeneous strategy still limits the spread of drug-resistance. This is because a spatially heterogeneous treatment maintains refuges for drug-sensitive parasites.

There is thus a critical migration range, above which the heterogeneous strategy may better limit the spread of drug-resistance. This critical migration range can be visualized in figure 2, at the interception point of the 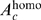 and 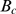 curves.

To illustrate further this point, let us take the example of two vector-borne diseases, malaria and trypanosomiasis. Even though we give here the example of two human diseases, recall that our models are general enough to be applicable to a wide range of parasites and hosts, including other animals and plants, provided the use of adequate parameters. Anopheles mosquitoes, malaria vectors, are known to migrate at longer ranges [47] than tsetse flies [48], which are responsible for the transmission of trypanosomiasis. Our model suggests that, because of the different migration patterns of their vectors, the optimal treatment strategy – treating everyone but not everywhere or treating everywhere but not everyone – that would best limit the spread of drug-resistance might differ between the two systems. It might be better to treat homogeneously against malaria (see figure 3a), while for trypanosomiasis the optimal strategy may depend on the available number of treatment doses (see figure 3b). Undoubtedly, treating only part of the population raises ethical questions. What appears to be the best solution for the population as a whole might not reveal immediately good for some individuals. As a result, untreated individuals looking for treatment may actively move towards treated areas, and may therefore mitigate the benefits of an heterogeneous treatment. Another way of creating a spatially heterogeneous environment for the parasite would be to use different drugs in different areas. This strategy, however, may select for multiple drug resistance (see [8] for a numerical investigation). Whether a spatial mosaic with two drugs better prevents the evolution of drug-resistance than a spatially homogeneous mixture of drugs remains to be investigated.

Of course, more quantitative recommendations for minimizing parasite prevalence and the evolution of drug-resistance would require a fully parameterized model of these two systems, as well as relaxing several simplifying assumptions. In particular, it would be worth extending our model to dispersal in two spatial dimensions, and taking diploidy and the effects of dominance into account. Both extensions have already been studied in a population genetics context by Nagylaki [22], who showed that the critical favorable area size is bigger in a model with two spatial dimensions, because the effect of migration is stronger [22]. Recessivity of the resistance locus also increases the critical favorable area size [22]. The analysis of these effects in models with a demographical feed-back on evolution requires further investigation. In the context of infectious diseases, it would also be interesting to study the potential influence of multiple infection events, whereby an already infected host can be infected by another strain: this would add another level of competition between parasitic strains, namely within-host competition. Finally, diffusion might not be the best way to model migration, especially in human populations, where individuals belong to interaction groups like households, and can be treated in specific locations like hospitals [9]. It would therefore be interesting to model more realistically both the spatial distribution of individuals (i.e. uneven distribution in space) as well as their migration patterns (i.e. various migration kernels).

In this paper we bridge the gap between the epidemiology and the population genetics of drug-resistance [49],[50] to study the interplay between demography and evolutionary dynamics in a spatially structured environment. Taking into account this eco-evolutionary feedback may help better predict and prevent the rise and spread of drug or vaccine resistance in pathogen populations.

## Materials and Methods

### Epidemiological models in a spatially heterogeneous environment

We study two models, corresponding to two types of parasite transmission. The parasites are modeled as asexual and haploid. In all models, the individuals are infected by one strain only at a time, and this strain cannot be displaced by the other (there is no coinfection or superinfection). No mutation is explicitly modeled: we assume that the drug-resistant strain pre-exists the treatment, and we study the outcome of competition with the resident strain. We assume that the hosts total density is constant in space and time. However, the total density of parasites varies.

We focus on simple spatial patterns of treatment: a pocket of treatment in an infinite untreated region (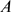 small compared to 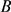, see figure 1b); or a periodical zebra-like pattern of treated and untreated regions (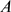 and 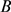 on the same scale, see figure 1c). The effect of treatment and the cost of resistance are represented in figure 1a using composite parameters, the basic reproductive ratios 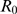 (see the main text).

The migration is modeled using the diffusion approximation. We assume that there is no directional preference (the mean of the dispersal kernel is zero), and that the standard deviation of the migration kernel is 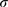 (the higher this parameter, the further dispersers go). Higher moments of the distribution are neglected. In the following, we present the direct transmission model; the analysis of the vector-borne transmission model is detailed in Text S1.

### Direct-transmission model

In our model with direct transmission of the parasites, only the hosts diffuse, independent of their infectious status; the parasites move with infected hosts. The densities of each parasite strain depend on time (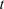) and space (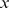). These changes can be written as a system of reaction-diffusion equations with three terms each (dropping the time and space dependency in 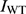 and 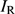 for readability):
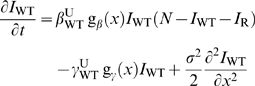
(14a)

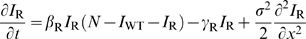
(14b)with 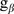 and 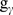 step functions that model the effects of the treatment, so that 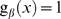 and 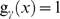 in the untreated area, and 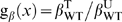 and 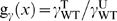 in the treated area. For each equation in system (14), the first term represents new infections with strain 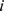, where the transmission of the disease from infected (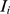) to uninfected (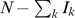) individuals happens at rate 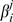. The second term is the recovery (or death) from the disease, equivalent to parasite clearance, which happens at rate 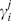. The last term stands for the diffusive migration of the hosts, with a migration range 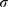, which is the standard deviation of the migration kernel.

The boundary conditions are periodic and reflecting:
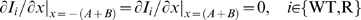
(15)It means that there is no net movement of individuals at the boundaries. Either the boundary cannot be crossed (like in a cage or on an island), or there is no net movement of individuals, because immigration and emigration compensate.

Let 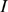 be the total density of infected individuals, and 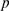 the proportion, among all infected individuals, of individuals infected by drug-resistant parasites:
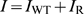
(16a)

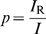
(16b)Using system (15), and with a little bit of algebra, we obtain the partial differential equations describing the dynamics of 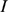 and 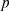, which are presented in the results section (see system (2)).

### Critical size in the direct transmission model

Finding the critical size of the treated (resp. untreated) area comes to studying the stability of the drug-resistant free (resp. drug-sensitive free) equilibrium. The method for the stability analysis with the direct transmission model, under the low migration approximation, is similar to the one already described in [34],[51],[52].

### Numerical Solutions

The sets of Partial Differential Equations (PDEs) can be numerically solved using the Method of Lines implemented in Mathematica's NDSolve function. For each set of parameters, two simulations are run, with different initial conditions, corresponding to the invasion of the drug-sensitive strain in an environment dominated by the drug-resistant strain, and reciprocally. If there are bistabilities, the ultimate outcomes of the two simulations are different.

## Supporting Information

Figure S1 Reciprocal Invasion Plot. It is the same plot as in figure 2 in the main text, but with different axes. Parameters: N = 100, β^U^_WT_ = 0.06, β_R_ = 0.055, β^T^_WT_ = 0.05, γ^U^_WT_ = 1, γ_R_ = 1.25, γ^T^_WT_ = 1.5
(0.20 MB TIF)

Figure S2 Reciprocal Invasion Plot. The parameters are different than in figure S1, and a fourth outcome is possible in zone (4). It corresponds to a situation where, at equilibrium, only one strain is maintained, but its type depends on the initial conditions. Parameters: N = 100, β^U^_WT_ = 0.05, β_R_ = 0.08, β^T^_WT_ = 0.09, γ^U^_WT_ = 1, γ_R_ = 2, γ^T^_WT_ = 2.5
(0.20 MB TIF)

Text S1 (0.25 MB PDF)
