# Supplementary material for: Evolutionary Epidemiology of Drug-Resistance in Space
Source: PLoS Comput Biol. 2009 Apr 3;5(4):e1000337. doi: 10.1371/journal.pcbi.1000337 (PMC2658742; doi:10.1371/journal.pcbi.1000337)
Supplement: Text S1 — (0.25 MB PDF) [file pcbi.1000337.s001.pdf]

Supplementary Information

for "Evolutionary Epidemiology of Drug-Resistance in Space"

Florence Débarre*, Thomas Lenormand*, Sylvain Gandon*

* Centre d'Ecologie Fonctionnelle et Evolutive, CNRS – UMR 5175, 1919 route de Mende, F-34293 Montpellier Cedex 5

Contents

1 Direct-transmission model **2**

1.1 Drug-resistant strain's invasion condition when $R_0^{\mathrm{WT,T}} > 1$ 2

1.2 Drug-resistant strain's invasion condition when $R_0^{\mathrm{WT,T}} < 1$ 4

1.3 Drug-sensitive strain's invasion condition 4

1.4 Homogeneous partial treatment 4

1.5 Feedback of demography on the evolution of resistance 6

2 Vector-borne transmission model **9**

2.1 Full Model 9

2.2 Compound parameters 10

2.3 Equivalent migration 10

2.4 Drug-resistant strain's invasion condition 11

2.5 Drug-sensitive strain's invasion condition 12

References **13**

1 Direct-transmission model

1.1 Drug-resistant strain's invasion condition when $R_0^{\mathrm{WT,T}} > 1$

We want to determine the condition of the size of the treated area, for drug-resistant parasites to invade a drug-sensitive parasite population.

To simplify the calculations, we focus on the part of the (periodic) environment located between $-A$ and B on the space axis, and assume that the area between $-A$ and 0 is treated.

If the migration range is low enough and $R_0^{\mathrm{WT,T}} > 1$, we can approximate the drug-resistant free equilibrium to:

$$\tilde{I}_{\mathrm{WT}}(x) = \begin{cases} N\,(1 - 1/R_0^{\mathrm{WT,T}}) & , \quad -A \leq x < 0 \\ N\,(1 - 1/R_0^{\mathrm{WT,U}}) & , \quad 0 < x \leq B \end{cases} \tag{A1a}$$

$$\tilde{I}_{\mathrm{R}}(x) = 0 \tag{A1b}$$

Linearizing equation (14b) in the main text around this equilibrium, we obtain:

$$\frac{\partial I_{\mathrm{R}}}{\partial t} = \begin{cases} \left(\frac{R_0^{\mathrm{R}}}{R_0^{\mathrm{WT,T}}} - 1\right)\gamma_{\mathrm{R}} I_{\mathrm{R}} + \frac{\sigma^2}{2}\frac{\partial^2 I_{\mathrm{R}}}{\partial x^2} & , \quad -A \leq x < 0 \\ \left(\frac{R_0^{\mathrm{R}}}{R_0^{\mathrm{WT,U}}} - 1\right)\gamma_{\mathrm{R}} I_{\mathrm{R}} + \frac{\sigma^2}{2}\frac{\partial^2 I_{\mathrm{R}}}{\partial x^2} & , \quad 0 < x \leq B \end{cases} \tag{A2}$$

with the boundary condititions:

$$\partial I_{\mathrm{R}}/\partial x|_{x=-A} = \partial I_{\mathrm{R}}/\partial x|_{x=B} = 0 \tag{A3}$$

and with the continuity conditions [1] (recall that $\tau = \dfrac{1 - 1/R_0^{\mathrm{WT,U}}}{1 - 1/R_0^{\mathrm{WT,T}}}$):

$$\tau \lim_{x \overset{\leq}{\to} 0} I_{\mathrm{R}} = \lim_{x \overset{\geq}{\to} 0} I_{\mathrm{R}} \tag{A4a}$$

$$\frac{1}{\tau} \lim_{x \overset{\leq}{\to} 0} \frac{\partial I_{\mathrm{R}}}{\partial x} = \lim_{x \overset{\geq}{\to} 0} \frac{\partial I_{\mathrm{R}}}{\partial x} \tag{A4b}$$

By separating the time and space scales [2, 3], we obtain the following system:

$$\frac{\partial^2 I_{\mathrm{R}}}{\partial x^2}(x) = -\frac{2}{\sigma^2}\left(\frac{R_0^{\mathrm{R}}}{R_0^{\mathrm{WT,T}}} - 1\right)\gamma_{\mathrm{R}} I_{\mathrm{R}}(x), \quad -A \leq x < 0 \tag{A5a}$$

$$\frac{\partial^2 I_{\mathrm{R}}}{\partial x^2}(x) = \frac{2}{\sigma^2}\left(1 - \frac{R_0^{\mathrm{R}}}{R_0^{\mathrm{WT,U}}}\right)\gamma_{\mathrm{R}} I_{\mathrm{R}}(x), \quad 0 < x \leq B \tag{A5b}$$

Solving system (A5) with the boundary (A3) and continuity (A4) conditions, we obtain the following constraint on the parameters:

$$\sqrt{\frac{2}{\sigma^2}\left(\frac{R_0^{\mathrm{R}}}{R_0^{\mathrm{WT,T}}} - 1\right)\gamma_{\mathrm{R}}}\, \tan\left[\sqrt{\frac{2}{\sigma^2}\left(\frac{R_0^{\mathrm{R}}}{R_0^{\mathrm{WT,T}}} - 1\right)\gamma_{\mathrm{R}}}\, A\right] = \tau^2 \sqrt{\frac{2}{\sigma^2}\left(1 - \frac{R_0^{\mathrm{R}}}{R_0^{\mathrm{WT,U}}}\right)\gamma_{\mathrm{R}}}\, \tanh\left[\sqrt{\frac{2}{\sigma^2}\left(1 - \frac{R_0^{\mathrm{R}}}{R_0^{\mathrm{WT,U}}}\right)\gamma_{\mathrm{R}}}\, B\right] \tag{A6}$$

solving equation (A6) for A, we recover the critical width of the treated area (see equation (8) in the main text):

$$A_c^{\mathrm{local}} = \frac{\sigma}{\sqrt{2}} \frac{1}{\sqrt{\gamma_{\mathrm{R}}}} \frac{1}{\sqrt{\frac{R_0^{\mathrm{R}}}{R_0^{\mathrm{WT,T}}} - 1}} \arctan\left(\tau^2 \sqrt{\frac{1 - \frac{R_0^{\mathrm{R}}}{R_0^{\mathrm{WT,U}}}}{\frac{R_0^{\mathrm{R}}}{R_0^{\mathrm{WT,T}}} - 1}}\, \tanh\left[\sqrt{\frac{2}{\sigma^2}\left(1 - \frac{R_0^{\mathrm{R}}}{R_0^{\mathrm{WT,U}}}\right)\gamma_{\mathrm{R}}}\, B\right]\right) \tag{A7}$$

The drug-resistant free equilibrium is stable when $A < A_c^{\mathrm{local}}$ [4]: the drug-resistant parasites cannot invade when the size of the treated area is below this critical size.

1.2 Drug-resistant strain's invasion condition when $R_0^{\mathrm{WT,T}} < 1$

When the migration range is small (low σ), we assume that the equilibrium density of a population fixed for the drug-sensitive allele is $N\,(1 - 1/R_0^{\mathrm{WT}})$ in the untreated area and 0 in the treated area (because $R_0^{\mathrm{WT,T}} < 1$). Using the method developed in [2, 3, 5], this leads to the following approximation for the critical size of the treated area:

$$A_c^{\mathrm{local(2)}} = \frac{\sigma}{\sqrt{2}} \frac{\pi}{2} \frac{1}{\sqrt{\gamma_{\mathrm{R}}}} \frac{1}{\sqrt{R_0^{\mathrm{R}} - 1}} \tag{A8}$$

1.3 Drug-sensitive strain's invasion condition

Using again the method developed in [2, 3], we find that the drug-sensitive parasites can only invade a population fixed for the drug-resistant parasites when the size of the untreated area is greater than a critical size B_c. This critical size reads:

$$B_c = \frac{\sigma}{\sqrt{2}} \frac{1}{\sqrt{\gamma_{\mathrm{WT}}^{\mathrm{U}}}} \frac{1}{\sqrt{\frac{R_0^{\mathrm{WT,U}}}{R_0^{\mathrm{R}}} - 1}} \arctan\left(\sqrt{\frac{\gamma_{\mathrm{WT}}^{\mathrm{T}}}{\gamma_{\mathrm{WT}}^{\mathrm{U}}}} \sqrt{\frac{1 - \frac{R_0^{\mathrm{WT,T}}}{R_0^{\mathrm{R}}}}{\frac{R_0^{\mathrm{WT,U}}}{R_0^{\mathrm{R}}} - 1}} \tanh\left[A \frac{\sqrt{2}}{\sigma} \sqrt{\gamma_{\mathrm{WT}}^{\mathrm{T}}} \sqrt{1 - \frac{R_0^{\mathrm{WT,T}}}{R_0^{\mathrm{R}}}} \right] \right) \tag{A9}$$

1.4 Homogeneous partial treatment

For comparison purposes, we have written a spatially homogeneous version of our one-host SIS-model (direct transmission model), where at each point in space a proportion $\rho = A/(A+B)$ of the population receives treatment, independent of the infectious status. We have to distinguish treated from untreated individuals, and therefore add compartments to the initial model ($I_{\mathrm{WT}} = I_{\mathrm{WT}}^{\mathrm{U}} + I_{\mathrm{WT}}^{\mathrm{T}}$). For the sake of readability, we drop the time and space dependency of the $I_{\mathrm{WT}}^{\mathrm{U}}$, $I_{\mathrm{WT}}^{\mathrm{T}}$ and I_{R} variables, and we note

$I = I_{\mathrm{WT}}^{\mathrm{U}} + I_{\mathrm{WT}}^{\mathrm{T}} + I_{\mathrm{R}}$, the total density of infected individuals. The equations read:

$$\frac{\partial I_{\mathrm{WT}}^{\mathrm{U}}}{\partial t} = (1-\rho)\,(\beta_{\mathrm{WT}}^{\mathrm{U}} I_{\mathrm{WT}}^{\mathrm{U}} + \beta_{\mathrm{WT}}^{\mathrm{T}} I_{\mathrm{WT}}^{\mathrm{T}})\,(N-I) - \gamma_{\mathrm{WT}}^{\mathrm{U}}\, I_{\mathrm{WT}}^{\mathrm{U}} + \frac{\sigma^2}{2}\frac{\partial^2 I_{\mathrm{WT}}^{\mathrm{U}}}{\partial x^2} \quad \text{(A10a)}$$

$$\frac{\partial I_{\mathrm{WT}}^{\mathrm{T}}}{\partial t} = \rho\,(\beta_{\mathrm{WT}}^{\mathrm{U}} I_{\mathrm{WT}}^{\mathrm{U}} + \beta_{\mathrm{WT}}^{\mathrm{T}} I_{\mathrm{WT}}^{\mathrm{T}})\,(N-I) - \gamma_{\mathrm{WT}}^{T}\, I_{\mathrm{WT}}^{T} + \frac{\sigma^2}{2}\frac{\partial^2 I_{\mathrm{WT}}^{\mathrm{T}}}{\partial x^2} \quad \text{(A10b)}$$

$$\frac{\partial I_{\mathrm{R}}}{\partial t} = \beta_{\mathrm{R}}\, I_{\mathrm{R}}\,(N-I) - \gamma_{\mathrm{R}}\, I_{\mathrm{R}} + \frac{\sigma^2}{2}\frac{\partial^2 I_{\mathrm{R}}}{\partial x^2} \quad \text{(A10c)}$$

The first equation gives the dynamics of untreated individuals infected by the drug-sensitive strain. The first term represents new infections: uninfected untreated individuals $(1-\rho)\,(N-I)$ are infected by individuals already infected by a drug-sensitive strain, at a rate β_{WT}^{j} which depends on the treatment status j ($j =$ U when untreated, $j =$T when treated) of the infecting individuals. Note that we make the simplifying assumption that the treatment may have an effect on disease transmissibility, but not on susceptibility to the disease. The second term is the recovery (or death), and the last term accounts for migration.

The second equation gives the dynamics of treated individuals infected by the drug-sensitive strain. The first term describes new infections: uninfected treated individuals $\rho\,(N-I)$ are infected by individuals infected by the drug-sensitive strain $(\beta_{\mathrm{WT}}^{\mathrm{U}} I_{\mathrm{WT}}^{\mathrm{U}} + \beta_{\mathrm{WT}}^{\mathrm{T}} I_{\mathrm{WT}}^{\mathrm{T}})$. The second term is recovery (or death) and the last term migration.

The last equation gives the dynamics of individuals infected by the drug-resistant strain. As the treatment has no effect on them, we do not have to distinguish between treated and untreated individuals.

Using the next generation method [6], we find the following basic reproductive ratios:

$$R_0^{\mathrm{WT}} = \frac{(1-\rho)N\beta_{\mathrm{WT}}^{\mathrm{U}}}{\gamma_{\mathrm{WT}}^{\mathrm{U}}} + \frac{\rho N \beta_{\mathrm{WT}}^{\mathrm{T}}}{\gamma_{\mathrm{WT}}^{\mathrm{T}}} = (1-\rho)R_0^{\mathrm{WT,U}} + \rho R_0^{\mathrm{WT,T}} \quad \text{(A11a)}$$

$$R_0^{\mathrm{R}} = \frac{N\beta_{\mathrm{R}}}{\gamma_{\mathrm{R}}} \quad \text{(A11b)}$$

The condition for the invasion of the drug-resistant strain is

$$R_0^{\mathrm{R}} > R_0^{\mathrm{WT}} \tag{A12}$$

The spatially heterogeneous strategy is such that all individuals are treated in a proportion $A/(A+B)$ of the environment. With the spatially homogeneous strategy presented here, the same proportion ($\rho = A/(A+B)$) of the total population is treated, but this proportion is the same everywhere in space. In order to compare the two strategies, we can rewrite equation (A12) as

$$A > A_c^{\mathrm{homo}} \tag{A13}$$

where

$$A_c^{\mathrm{homo}} = B\frac{R_0^{\mathrm{WT,U}} - R_0^{\mathrm{R}}}{R_0^{\mathrm{R}} - R_0^{\mathrm{WT,T}}} \tag{A14}$$

This expression with the basic reproductive ratios is also valid for the vector-borne transmission.

1.5 Feedback of demography on the evolution of resistance

To illustrate the effect of the feedback of demography on the evolution of resistance, we contrast the results of our direct transmission model, which is an epidemiogical model (see equation 2b in the main text) with a model where the parasite population size is constrained to be constant over space [4]. This model without demographical feedback reads:

$$\frac{\partial p}{\partial t} = S(x)\, p\,(1-p) + \frac{\sigma^2}{2}\frac{\partial^2 p}{\partial x^2} \tag{A15}$$

where $S(x)$ is a fitness function, such that, for $-(A+B) < x < A+B$ ($_{ND}$ standing

for non demographic):

$$S(x) = \begin{cases} s_{ND} = N\left(\beta_{\mathrm{R}} - \beta^{\mathrm{T}}_{\mathrm{WT}}\right) - \left(\gamma_{\mathrm{R}} - \gamma^{\mathrm{T}}_{\mathrm{WT}}\right) & |x| < A \\ -\alpha_{ND}^2\, s_{ND} = N\left(\beta_{\mathrm{R}} - \beta^{\mathrm{U}}_{\mathrm{WT}}\right) - \left(\gamma_{\mathrm{R}} - \gamma^{\mathrm{U}}_{\mathrm{WT}}\right) & |x| \geq A \end{cases} \tag{A16}$$

$S(x)$ in equation (A16) is equivalent to $\bar{s}(I, x)$ (equation (5) in the main text) being evaluated for I close to zero, which corresponds to an initial growth rate.

Nagylaki [4] derived the critical size of the area favorable to the allele of interest (i.e. in our context, the treated area):

$$A_c^{Nagylaki} = \frac{\sigma}{\sqrt{2\, s_{ND}}} \arctan\left(\alpha_{ND} \tanh\left[\alpha_{ND}\sqrt{s_{ND}}\frac{\sqrt{2}}{\sigma}B\right]\right) \tag{A17}$$

In figure A1 we compare the models with and without demographical feedback. We plot the equilibrium frequency of drug-resistance for both models. With the set of parameters used in figure A1, drug-resistance appears for a smaller proportion of treated individuals in the model without demographical feedback (8% instead of 14%). This is because the initial asymmetry in population densities in the epidemiological model reinforces the migration of sensitive parasites into the treated area, which hinders the rise of drug-resistant parasites.

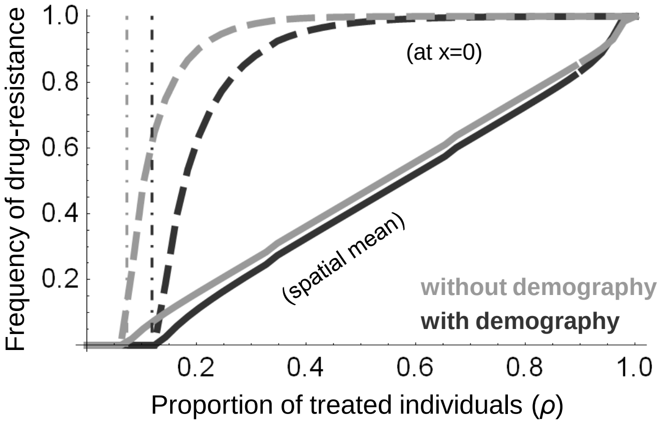

Figure A1: Comparison between with (darkgray) and without (lightgray) demographical feedback. The frequency of resistance depends on the proportion of treated individuals. The full curves show the spatial mean of the frequency of resistance, and the dashed curves the maximal frequency, at $x = 0$. The vertical dot-dashed lines show the analytical critical proportions of treated individuals, which are derived from equation (8) in the main text, and equation (A17) here. Parameters: $(A + B)/\sigma = 10$,$N = 100$, $\beta^{\mathrm{T}}_{\mathrm{WT}} = 0.02$, $\beta^{\mathrm{U}}_{\mathrm{WT}} = 0.06$, $\beta_{\mathrm{R}} = 0.03$, $\gamma^{\mathrm{T}}_{\mathrm{WT}} = 1.25$, $\gamma^{\mathrm{U}}_{\mathrm{WT}} = 1$, $\gamma_{\mathrm{R}} = 1.2$ ($R_0^{\mathrm{WT,U}} = 6$, $R_0^{\mathrm{WT,T}} = 1.6$, $R_0^{\mathrm{R}} = 2.5$)

2 Vector-borne transmission model

2.1 Full Model

The full model reads:

$$\frac{\partial I_{\mathrm{WT}}}{\partial t} = \beta^{\mathrm{U}}_{\mathrm{WT}} g_\beta(x)\, V_{\mathrm{WT}}\, (N_{\mathrm{H}} - I_{\mathrm{WT}} - I_{\mathrm{R}}) - \gamma^{\mathrm{U}}_{\mathrm{WT}} g_\gamma(x)\, I_{\mathrm{WT}} + \frac{\sigma_{\mathrm{H}}^2}{2}\frac{\partial^2 I_{\mathrm{WT}}}{\partial x^2} \tag{A18a}$$

$$\frac{\partial V_{\mathrm{WT}}}{\partial t} = b^{\mathrm{U}}_{\mathrm{WT}}\, g_b(x)\, I_{\mathrm{WT}}\, (N_{\mathrm{V}} - V_{\mathrm{WT}} - V_{\mathrm{R}}) - \nu^{\mathrm{U}}_{\mathrm{WT}}\, g_\nu(x)\, V_{\mathrm{WT}} + \frac{\sigma_{\mathrm{V}}^2}{2}\frac{\partial^2 V_{\mathrm{WT}}}{\partial x^2} \tag{A18b}$$

$$\frac{\partial I_{\mathrm{R}}}{\partial t} = \beta_{\mathrm{R}}\, V_{\mathrm{R}}\, (N_{\mathrm{H}} - I_{\mathrm{WT}} - I_{\mathrm{R}}) - \gamma_{\mathrm{R}}\, I_{\mathrm{R}} + \frac{\sigma_{\mathrm{H}}^2}{2}\frac{\partial^2 I_{\mathrm{R}}}{\partial x^2} \tag{A18c}$$

$$\frac{\partial V_{\mathrm{R}}}{\partial t} = b_{\mathrm{R}}\, I_{\mathrm{R}}\, (N_{\mathrm{V}} - V_{\mathrm{WT}} - V_{\mathrm{R}}) - \nu_{\mathrm{R}}\, V_{\mathrm{R}} + \frac{\sigma_{\mathrm{V}}^2}{2}\frac{\partial^2 V_{\mathrm{R}}}{\partial x^2} \tag{A18d}$$

Equations (A18a) and (A18c) describe the dynamics of the infected humans (I), where the first term represents new infections with strain i, happening at rate β_i, when uninfected hosts ($N_{\mathrm{H}} - \sum_k I_k$) are bitten by infected vectors V_i; the second term stands for host recovery (or death); the last term represent hosts' migration, at range σ_{H}. Equations (A18b) and (A18d) describe the dynamics of the vectors (V), where the first term represents new infections, which happen at rate b_i when uninfected vectors ($N_{\mathrm{V}} - \sum_k V_k$) bite infected humans I_i; the second term represents the vectors' death, at rate ν_i – corresponding to parasite clearance –; the last term accounts for vectors' migration, at range σ_{V}. Note the total vector density N_{V} is constant, like the total human density N_{H}.

Like in the direct-transmission model, the boundary conditions are periodic and reflecting.

2.2 Compound parameters

The basic reproductive ratios of strain i in area j with the vector-borne transmission are:

$$R_0^{i,j} = \frac{N_{\text{H}} N_{\text{V}} \beta_i^j b_i^j}{\gamma_i^j \nu_i^j} \tag{A19}$$

The initial asymmetries in the densities of the drug sensitive strain between habitats are summarized in τ_{H} (for humans) and τ_{V} (for vectors):

$$\tau_{\text{H}} = \frac{\tilde{I}^{\text{U}}_{\text{WT}}}{\tilde{I}^{\text{T}}_{\text{WT}}} \tag{A20a}$$

$$\tau_{\text{V}} = \frac{\tilde{V}^{\text{U}}_{\text{WT}}}{\tilde{V}^{\text{T}}_{\text{WT}}} \tag{A20b}$$

where

$$\tilde{I}^j_{\text{WT}} = \frac{N_{\text{H}} N_{\text{V}} b^j_{\text{WT}} \beta^j_{\text{WT}} - \gamma^j_{\text{WT}} \nu^j_{\text{WT}}}{b^j_{\text{WT}}(N_{\text{V}} \beta^j_{\text{WT}} + \gamma^j_{\text{WT}})} \tag{A21a}$$

$$\tilde{V}^j_{\text{WT}} = \frac{N_{\text{H}} N_{\text{V}} b^j_{\text{WT}} \beta^j_{\text{WT}} - \gamma^j_{\text{WT}} \nu^j_{\text{WT}}}{\beta^j_{\text{WT}}(N_{\text{H}} b^j_{\text{WT}} + \nu^j_{\text{WT}})} \tag{A21b}$$

2.3 Equivalent migration

In a two-host model, the parasite must infect sequentially the host and its vector in order to complete its life-cycle. Both host and vector can migrate and therefore let the parasite move spatially. The human's (resp. vector's) infection duration is $1/\gamma_{\text{R}}$ (resp. $1/\nu_{\text{R}}$) when infected by a drug-resistant strain. Consequently, the parasite spends a proportion $q = \dfrac{1/\gamma_{\text{R}}}{1/\nu_{\text{R}} + 1/\gamma_{\text{R}}}$ of its life-cycle in the human compartment. This is equivalent to a species which would migrate with a parameter σ_{H} during a proportion q of its life-cycle, and with a parameter σ_{V} during the remaining $1 - q$. The temporal

change in density X of this species, due to migration only, is

$$\begin{aligned}\frac{\partial X}{\partial t} &= q\,\frac{\sigma_{\mathrm{H}}^2}{2}\frac{\partial^2 X}{\partial x^2} + (1-q)\,\frac{\sigma_{\mathrm{V}}^2}{2}\frac{\partial^2 X}{\partial x^2} \\ &= \frac{\sigma_e^2}{2}\frac{\partial^2 X}{\partial x^2}\end{aligned} \tag{A22}$$

where

$$\sigma_e^2 = \frac{\sigma_{\mathrm{V}}^2/\nu_{\mathrm{R}} + \sigma_{\mathrm{H}}^2/\gamma_{\mathrm{R}}}{1/\nu_{\mathrm{R}} + 1/\gamma_{\mathrm{R}}} \tag{A23}$$

2.4 Drug-resistant strain's invasion condition

Linearizing around the equilibrium corresponding to a wholly drug sensitive parasite population, we obtain the following system (where $k = \mathrm{T}$ in the treated area, and $k = \mathrm{U}$ in the untreated):

$$\frac{\partial}{\partial t}\begin{pmatrix} I_{\mathrm{R}} \\ V_{\mathrm{R}} \end{pmatrix} = M_k \begin{pmatrix} I_{\mathrm{R}} \\ V_{\mathrm{R}} \end{pmatrix} + \frac{1}{2}\begin{pmatrix} \sigma_{\mathrm{H}}^2 \\ \sigma_{\mathrm{V}}^2 \end{pmatrix} \frac{\partial^2}{\partial x^2}\begin{pmatrix} I_{\mathrm{R}} \\ V_{\mathrm{R}} \end{pmatrix} \tag{A24}$$

where

$$M_k = \begin{pmatrix} -\gamma_{\mathrm{WT}}^k & \dfrac{\beta_{\mathrm{WT}}^k \gamma_{\mathrm{R}}(N_{\mathrm{H}} b_{\mathrm{R}} + \nu_{\mathrm{R}})}{b_{\mathrm{R}}(N_{\mathrm{V}}\beta_{\mathrm{R}} + \gamma_{\mathrm{R}})} \\ \dfrac{b_{\mathrm{WT}}^k \nu_{\mathrm{R}}(N_{\mathrm{V}}\beta_{\mathrm{R}} + \gamma_{\mathrm{R}})}{\beta_{\mathrm{R}}(N_{\mathrm{H}} b_{\mathrm{R}} + \nu_{\mathrm{R}})} & -\nu_{\mathrm{WT}}^k \end{pmatrix} \tag{A25}$$

Let λ_{T} (resp. λ_{U}) be the dominant eigenvalue of M_{T} (resp. M_{U}):

$$\lambda_k = \frac{1}{2}\left(\sqrt{4\frac{b_{\mathrm{R}}\beta_{\mathrm{R}}\gamma_{\mathrm{WT}}^k\nu_{\mathrm{WT}}^k}{\beta_{\mathrm{WT}}^k b_{\mathrm{WT}}^k} + (\gamma_{\mathrm{R}} - \nu_{\mathrm{R}})^2} - (\gamma_{\mathrm{R}} + \nu_{\mathrm{R}})\right) \tag{A26}$$

System (A24) can be approximated as:

$$\frac{\partial}{\partial t}\begin{pmatrix} I_{\mathrm{R}} \\ V_{\mathrm{R}} \end{pmatrix} = \lambda_k \begin{pmatrix} I_{\mathrm{R}} \\ V_{\mathrm{R}} \end{pmatrix} + \frac{1}{2}\,\sigma_e\,\frac{\partial^2}{\partial x^2}\begin{pmatrix} I_{\mathrm{R}} \\ V_{\mathrm{R}} \end{pmatrix} \tag{A27}$$

If we assume that selection is weak, we get the following approximation:

$$\lambda_k \approx \left(\frac{R_0^{\text{R}}}{R_0^{\text{WT},k}} - 1 \right) \frac{1}{1/\gamma_{\text{R}} + 1/\nu_{\text{R}}} \tag{A28}$$

Then, the same method as with the one-host model is used, with $s = \lambda_{\text{T}}$ and $\alpha = \sqrt{-\lambda_{\text{U}}/\lambda_{\text{T}}}$. We however could not find a single equivalent for τ (see system (A20) above), and therefore obtain two critical sizes of the treated area (A_c^{H} and A_c^{V}), depending on whether the initial drug-resistant free equilibrium densities are evaluated in the human or vector compartments.

2.5 Drug-sensitive strain's invasion condition

We use the same method as above, and find that the drug-sensitive parasites can invade a drug-resistant parasite population when the size of the untreated area, B, is above a critical size B_c. This critical size reads:

$$B_c = \frac{\sigma_e}{\sqrt{2}} \frac{1}{\sqrt{\lambda'_{\text{U}}}} \arctan \left(\sqrt{-\frac{\lambda'_{\text{T}}}{\lambda'_{\text{U}}}} \tanh \left[\sqrt{-\lambda'_{\text{T}}} \frac{\sqrt{2}}{\sigma_e} A \right] \right) \tag{A29}$$

with ($k =$T or $k =$U)

$$\lambda'_k = \frac{1}{2} \left(\sqrt{\frac{4\, b_{\text{WT}}^k \beta_{\text{WT}}^k\, \gamma_{\text{R}} \nu_{\text{R}}}{b_{\text{R}} \beta_{\text{R}}} + (\gamma_{\text{WT}}^k - \nu_{\text{WT}}^k)^2} - (\gamma_{\text{WT}}^k + \nu_{\text{WT}}^k) \right) \tag{A30}$$

References

[1] Nagylaki T (1978) Clines with asymmetric migration. Genetics 88:813–827.

[2] Pacala SW, Roughgarden J (1982) Spartial Heterogeneity and Interspecific Competition. Theor Popul Biol 21:92–113.

[3] Nuismer SL, Thompson JN, Gomulkiewicz R (2000) Coevolutionary clines across selection mosaics. Evolution 54:1102–1115.

[4] Nagylaki T (1975) Conditions for the existence of clines. Genetics 80:595–615.

[5] Sasaki T (2004) The effect of local prevention in an SIS model with diffusion. Discrete Cont Dyn-B 4:739–746.

[6] Heffernan JM, Smith RJ, Wahl LM (2005) Perspectives on the basic reproductive ratio. J R Soc Interface 2:281–293. doi:10.1098/rsif.2005.0042. URL http://dx.doi.org/10.1098/rsif.2005.0042.
